# Supplementary material for: Antigen heterogeneity in the development and clinical validation of a multiplexed urine test for tuberculosis
Source: Commun Med (Lond). 2026 Mar 10;6:219. doi: 10.1038/s43856-026-01458-0 (PMC13076864; doi:10.1038/s43856-026-01458-0)
Supplement: Supplementary file 2 — Supplementary Information [file 43856_2026_1458_MOESM2_ESM.docx]

Antigen heterogeneity in the development and clinical validation of a multiplexed urine test for tuberculosis

Supplementary Information

**Authors:**

Tyler J. Dougan^†1,2,3,4,5^, Shira Roth^†1,2,3^, Liangxia Xie^1,2,3^, Sydney D’Amaddio^1,2,3^, David R. Walt^*1,2,3^

**Affiliations:**

^1^ Wyss Institute for Biologically Inspired Engineering, Harvard University; Boston, MA 02115, USA.

^2^ Department of Pathology, Brigham and Women's Hospital; Boston, MA 02115, USA.

^3^ Harvard Medical School, Harvard University; Boston, MA 02115, USA.

^4^ Harvard-MIT Program in Health Sciences and Technology, Massachusetts Institute of Technology; Cambridge, MA 02139, USA.

^5^ Present address: Africa Health Research Institute, Durban, South Africa.

*Corresponding author. Email: [dwalt@bwh.harvard.edu](mailto:dwalt@bwh.harvard.edu).

† These authors contributed equally to this work

**Materials and Methods:**

Single Molecule Array assays

Assay development

Ag85B pull-down and silver staining

**Figure S1.** Calibration curves of all investigated markers.

**Figure S2.** Cross-testing LAM antibodies.

**Figure S3.** No cross-reactivity between the assays in dropout experiments.

**Figure S4.** Dilution linearity.

**Figure S5.** Admixture linearity.

**Figure S6.** Ag85B capture antibody (182λ) identifies the native form of Ag85B.

**Figure S7.** Spike and recovery of LAM and Ag85B.

**Figure S8.** Recovery of Ag85B is negatively affected by urea in the urine and is dependent on urine total protein.

**Figure S9.** Urea attenuates Ag85B concentration and spans a wide range in urine samples.

**Figure S10.** Attempts to improve recovery. Adding urea to the spiked matrix slightly improved the recovery of Ag85B.

**Figure S11.** Flow diagram of model training and evaluation.

**Figure S12.** Partial dependence plots for the model evaluated on the test cohort.

**Figure S13.** AUC-ROC scores with different combinations of biomarkers.

**Table S1.** Assay conditions used for the 11-marker panel.

**Table S2.** TB biomarker Simoa assay characteristics.

**Table S3.** Detectability of 11 TB markers in the discovery cohort.

**Table S4.** Characteristics of common TB diagnostic tests.

**Table S5.** TB categories and classifications.

**Table S6.** AlereLAM results using 244 samples from the blinded test set.**Single Molecule Array assays**

Simoa is based on sandwich enzyme-linked immunosorbent assays (ELISAs), which use the selectivity of a pair of antibodies to quantify an analyte macromolecule ^29^. In Simoa, these reactions take place on beads, so that the signal from a single binding event can be detected. Paramagnetic beads are coated with a capture antibody, and these beads are added to the solution to be tested. Because hundreds of thousands of beads are added to a relatively small volume, mass transport to the bead surface is rapid. For low-abundance measurements (fM–aM), there are many more beads than molecules, so that after binding most beads contain no molecules and some contain a single bound molecule. Beads are washed to remove interfering molecules, the detection antibody is added, and an enzyme is bound to the detection antibody. After washing, the only enzymes left are those attached to a bead with an analyte molecule bound. The beads are loaded into wells with the enzyme’s substrate, which the enzyme converts into a fluorescent product. A camera images and counts the number of fluorescent wells (meaning they have a protein molecule bound) and the total number of wells containing a bead. These values are used to calculate the average enzyme per bead (AEB), which is converted back to a concentration by generating a calibration curve with different dilutions of a purified form of the analyte. Whereas traditional ELISA uses a bulk measurement of total fluorescence, Simoa isolates beads into individual wells, and because there are many more beads than analyte molecules, the number of molecules can be calculated from the number of fluorescent beads using Poisson statistics.

*Bead conjugation*—Capture antibodies were reconstituted or buffer exchanged into 50 mM 2-(N-morpholino)ethanesulfonic acid (MES) buffer (Quanterix, Billerica, MA) via three washes through a 50 kDa Amicon Ultra-0.5 centrifugal filter according to the manufacturer’s instructions. Recovered antibodies were diluted to 0.2 mg/mL in 50 mM MES. A volume of 2.5 μm carboxylated paramagnetic beads (Quanterix, Billerica, MA) containing 1.4 × 10^9^ beads per mL of diluted antibody was transferred to a new microcentrifuge tube. Beads were washed by placing the microcentrifuge tube on a magnetic separator, waiting for the beads to collect, aspirating the supernatant, removing the tube from the magnetic separator, adding fresh buffer, and vortexing to mix. Beads were washed three times in Bead Wash Buffer (Phosphate-buffered saline (PBS) with Tween 20, Quanterix, Billerica, MA) and three times in 50 mM MES (Quanterix, Billerica, MA). Carboxyl groups on the beads’ surfaces were then activated by incubating in 0.3 mg/mL 1-Ethyl-3-(3-dimethylaminopropyl)carbodiimide (EDC) (Thermo Fisher) in 50 mM MES for 30 minutes at 4 ºC, with gentle shaking to keep them in suspension. Beads were washed once with 50 mM MES and then incubated with the 0.2 mg/mL capture antibody for 2 hours at 4 ºC, with gentle shaking to keep them in suspension. Beads were washed twice in Bead Wash Buffer and blocked by incubating in Bead Blocking Buffer (PBS with Bovine Serum Albumin (BSA), Quanterix, Billerica, MA) for 45 min at room temperature or overnight at 4 ºC. Finally, beads were washed once with Bead Wash Buffer, once with Bead Diluent (Tris-buffered saline (TBS) with Tween 20 and BSA, Quanterix, Billerica, MA), resuspended in Bead Diluent, and counted using a Coulter Counter. The typical batch volume was 300 μL (4.2 × 10^8^ beads), but volumes were adjusted proportionally for larger and smaller batches.

*Detector biotinylation*—Detection antibodies were reconstituted or buffer exchanged into PBS (Quanterix, Billerica, MA) via three washes through a 50 kDa Amicon Ultra-0.5 centrifugal filter according to the manufacturer’s instructions. Recovered antibodies were diluted to 1 mg/mL in PBS. NHS-PEG4-biotin (N-Hydroxysuccinimide (NHS)-polyethylene glycol 4 (PEG4)-biotin ,Thermo Fisher) was diluted to 8.9 mM in deionized water (2 mg NHS-PEG4-biotin / 383 μL water) and added to detection antibody at a concentration of 0.267 mM (0.157 mg/mL), corresponding to a 40-fold molar ratio of biotin to antibody monomer. In a typical batch, 3 μL of 8.9 mM biotin were added to 100 μL of antibody. After mixing, the solution was incubated for 30 minutes at room temperature. Excess biotin was removed in three washes of PBS through a 50 kDa Amicon Ultra-0.5 centrifugal filter according to the manufacturer’s instructions, and the final antibody concentration was determined with a NanoDrop spectrophotometer.

*Simoa assay*—A Quanterix HD-X Analyzer automatically performed the 3-step Simoa assay at room temperature. First, 170 μL of each diluted sample or calibrator was combined with 25 μL of bead reagent in a reaction cuvette and incubated for 30 minutes with shaking to keep the beads in suspension. The bead reagent consisted of 5 × 10^6^ beads/mL of equal parts each of the four plexes of beads (one for Ag85B and three for LAM, as shown in Figure 1, 1.25 × 10^6^ beads/mL for each plex) in a bead diluent of 50 mM TBS with 2% BSA, 0.1% Tergitol (NP-40, MilliporeSigma), and 0.03% ProClin 300. The beads were drawn to the side of the cuvette with a magnet and washed several times with System Wash Buffer 1 (5X PBS with Tween 20, Quanterix) to remove unbound sample. In the second step, beads were incubated for 5 minutes in 100 μL of detector reagent, consisting of 0.6 μg/mL of biotinylated Ag85B antibody 149 and 0.1 μg/mL of biotinylated LAM antibody A194-01 IgM in the detector diluent: PBS with 2% BSA, 5 mM EDTA, 0.1% Tergitol, and 0.03% ProClin 300. In the third step, after several washes with System Wash Buffer 1 to remove unbound detection antibodies, the beads were incubated for 5 minutes in 100 μL of streptavidin-β-galactosidase (SBG) reagent, consisting of 100 pM SBG in SBG Diluent (PBS with BSA, Tween 20, ProClin 300, and an interference blocker, Quanterix). Finally, excess SBG was washed away using Wash Buffer 1 followed by PBS (Quanterix) to remove Tween 20, and the beads were resuspended in 25 μL of resorufin-β-D-galactopyranoside (RGP)and loaded into an array of femtoliter-sized wells. The wells were sealed with oil and imaged using a fluorescence microscope. In wells containing beads with SBG bound (“on” beads), the SBG converted RGP to a fluorescent product, resorufin, that was visible in the 574/615 nm image. The images were automatically analyzed by the HD-X image processing software to identify which wells contained beads, categorize beads by plex (fluorescent channel), and measure the intensity of each well in the resorufin channel. For each sample, it calculated *f*_on_, the fraction of beads with increased intensity in the resorufin channel; *I*_bead_, the average resorufin intensity of “on” beads; and the average number of enzymes per bead (AEB) according to digital ELISA (using Poisson statistics) and analog ELISA (based on average intensity).

**Assay development and validation**

Simoa assays for 11 *M. tb* antigens in urine (Fig S12, Table S4, Table S5) were validated through dilution linearity; spike and recovery; and dropout tests. In dilution linearity, urine samples from three individuals with TB were successively diluted neat, 1:2, 1:4, and 1:8 in sample diluent to confirm that for each two-fold increase in dilution factor, the measured concentration decreased by a factor of two. In spike and recovery, urine samples from three individuals without TB were spiked with known concentrations of analyte standard, and the measured (recovered) concentration was compared to the known (spiked) concentration. In dropout tests, only one of the standards (LAM or Ag85B) was added each time to evaluate its effect on the measured concentration of the other biomarker.

To find the best antibody pairs for each target, a pairwise screening process was conducted using the HD-X analyzer and a pooled set of positive TB samples from the training cohort diluted with healthy urine. Available antibodies were paired as either capture reagents (conjugated to beads) or detection reagents (biotinylated). The antibodies were screened in a three-step format, and the SNR values were obtained. Optimal pairs showed a high signal-to-background ratio with a low background signal (Figure S2). A194 as a detector gave the highest SNR across most antibodies tested. A194-01 IgM (Rutgers University, Newark, NJ, USA) is best characterized and was shown previously to bind to urine LAM as a detector antibody (20, 22). Therefore, in addition to the antibody screening, other considerations when choosing the final antibodies included the reproducibility of the antibodies, the epitopes the antibodies bind to, and the isoelectric point (pI) of the antibodies. Considering all these parameters, the A194-01 was chosen as the detector for our LAM assays.

The capture antibodies chosen for LAM were G3, S4-20 (Otsuka Pharmaceutical Co., Tokyo, Japan), and FIND28 (FIND). The capture antibody chosen for Ag85B was the 182λ antibody, and the detector was the 149 antibody. The Ag85B antibodies were obtained from AbCellera Biologics (Vancouver, Canada) under material transfer agreements for Simoa assay development. Genscript was contracted to produce large batches of identified antibodies using sequence information provided by AbCellera Biologics under non-disclosure agreements.

A four-plex Simoa assay was developed and optimized by adjusting various assay parameters, including detection antibodies concentration, enzyme concentration, assay format, incubation times, and diluent buffers.

**Table S1.** Assay conditions used for the 11-marker panel during assay development and testing, as compared with final assay conditions used for samples. Abbreviations: LOD: limit of detection; TBST: tris-buffered saline with Tween 20, pH 8.0; SD: Quanterix General Sample/Detector Diluent; SU: synthetic urine. All non-LAM antibody clones were developed by AbCellera and produced by GenScript as described in the main text. All other preliminary assay conditions were the same as described in the Methods and Supplemental Methods except for the bead diluent (Quanterix Bead Diluent), detector diluent (Quanterix General Sample/Detector Diluent), Simoa diluted sample reaction volume (100 μL), capture incubation time (15 minutes for 3-step assay or 30 minutes for a 2-step assay), SBG concentration (150 pM), and RGP volume (50 μL).

|  | **Capture** **Clone** | | **Detector**  **Clone** | | **Detector**  **(μg/mL)** | **Standard**  **Source** | **No. Plexes** | **Assay**  **Steps** | **Sample**  **Diluent** | **Dilution**  **Factor** | **Calibrator**  **Diluent** | **Assay**  **Beads/mL** |
| --- | --- | --- | --- | --- | --- | --- | --- | --- | --- | --- | --- | --- |
| *11-marker panel assay conditions* | | | | | |  |  |  |  |  |  |  |
| Ag85A | | 285 | | 164 | 0.3 | AbCellera | 4 | 3 | SD | 3:4 | Ricca SU | 1.5 × 10^6^ |
| Ag85B | | 182 | | 149 | 0.3 | AbCellera |  |  |  |  |  | 1.5 × 10^6^ |
| Ag85C | | 166 | | 187 | 0.3 | AbCellera |  |  |  |  |  | 1.5 × 10^6^ |
| Adk | | 13 | | 11 | 0.3 | AbCellera |  |  |  |  |  | 1.5 × 10^6^ |
| GlcB | | 224 | | 214 | 0.17 | AbCellera | 2 | 2 | SD | 3:4 | Ricca SU | 2.5 × 10^6^ |
| RelG | | 310 | | 370 | 0.1 | AbCellera |  |  |  |  |  | 2.5 × 10^6^ |
| GroES | | 256 | | 249 | 0.3 | SomaLogic | 1 | 3 | TBST | 3:4 | Ricca SU | 2.5 × 10^6^ |
| CFP10 | | 272 | | 44 | 0.17 | BEI | 1 | 2 | SD | 3:4 | BioIVT SU | 2.5 × 10^6^ |
| Mce1A | | 341 | | 474 | 0.17 | AbCellera | 1 | 2 | SD | 1:2 | Sigmatrix SU | 2.5 × 10^6^ |
| HspX | | 220 | | 195 | 0.13 | FIND | 1 | 2 | SD | 3:4 | Ricca SU | 2.5 × 10^6^ |
| LAM | | S4-20 | | A194 | 0.6 | BEI | 1 | 3 | SD | 1:2 | SD | 2.5 × 10^6^ |
| *Final assay conditions* | | | | | |  |  |  |  |  |  |  |
| Ag85B | | 182 | | 149 | 0.6 | BEI | 4 | 3 | 2X PBS, 4% BSA, 10 mM EDTA, 0.06% ProClin 300 | 1:2 | 1X PBS,  2% BSA, 5 mM EDTA, 0.03% ProClin 300 | 1.25 × 10^6^ |
| LAM | | S4-20 |  | A194 | 0.1 | BEI |  |  |  |  |  | 1.25 × 10^6^ |
|  |  | FIND28 | |  |  |  |  |  |  |  |  | 1.25 × 10^6^ |
|  |  | G3 |  |  |  |  |  |  |  |  |  | 1.25 × 10^6^ |

**Figure S1.** Calibration curves of all investigated markers. AEB is the signal unit for Simoa measurements. AEB: average enzyme per bead.

**Table S2.** TB biomarker Simoa assay characteristics. LOD: limit of detection.

| **Marker** | **LOD (pg/mL)** | **Average Recovery %**  **(min-max)** | **Average Dilution Linearity Fitted R^2^** |
| --- | --- | --- | --- |
| Ag85A | 0.017 | 107 (92-130) | >0.99 |
| Ag85B | 0.015 | 109 (86-146) | 0.99 |
| Ag85C | 0.001 | 114 (95-138) | >0.99 |
| Adk | 0.16 | 111 (70-175) | 0.99 |
| GroES | 0.015 | 101 (75-141) | 0.98 |
| GlcB | 0.025 | 109 (82-135) | >0.99 |
| RelG | 0.004 | 119 (99-168) | >0.99 |
| LAM | 5.91 | 90 (70-113) | 0.98 |
| CFP10 | 0.56 | 86 (52-155) | 0.98 |
| Mce1A | 0.70 | 94 (36-162) | 0.94 |
| HspX | 0.069 | 88 (19-162) | >0.99 |

**Table S3.** Detectability of 11 TB markers in the discovery cohort. Data are n (%). A one-sided Mann-Whitney U test was used to test the significance of the difference between TB and non-TB patients with Bonferroni correction for multiple comparisons. Undetectable samples were assigned a value at the assay limit of detection (LOD) to be included in the analysis.

| **Antigen** | **LOD (pg/mL)** | **Number of detectable samples** | | **p value** | **Include/Exclude**  **for model construction** |
| --- | --- | --- | --- | --- | --- |
|  |  | TB (n=55) | Non-TB (n=45) |  |  |
| LAM | 5.91 | 53 (96%) | 13 (29%) | <0.0001 | Include |
| Ag85B | 0.015 | 45 (82%) | 19 (42%) | <0.0001 | Include |
| Ag85C | 0.001 | 37 (67%) | 22 (49%) | 0.094 | Exclude |
| Ag85A | 0.017 | 13 (24%) | 22 (49%) | - | Exclude |
| Adk | 0.16 | 9 (16%) | 13 (29%) | - | Exclude |
| GroES | 0.015 | 6 (11%) | 4 (9%) | - | Exclude |
| GlcB | 0.025 | 2 (4%) | 6 (13%) | - | Exclude |
| RelG | 0.004 | 5 (9%) | 8 (18%) | - | Exclude |
| CFP10 | 0.56 | 15 (27%) | 7 (16%) | - | Exclude |
| Mce1A | 0.70 | 6 (11%) | 9 (20%) | - | Exclude |
| HspX | 0.069 | 1 (2%) | 1 (2%) | - | Exclude |

**Figure S2**. Cross-testing LAM antibodies. The values represent the signal-to-background ratios in a Simoa measurement where signal was measured using diluted pooled urine from individuals with TB and background using diluted pooled urine from individuals without TB, all from the training cohort. This allowed one pooled set to be standardized across all antibody pairs, and the dilution accounts for the low signal-to-background ratios seen even with the best pairs. Each row represents one capture antibody; each column represents one detection antibody. Pairs that were not measured are indicated in white. These results were combined with findings from the literature to select the antibody pairs for the final assay


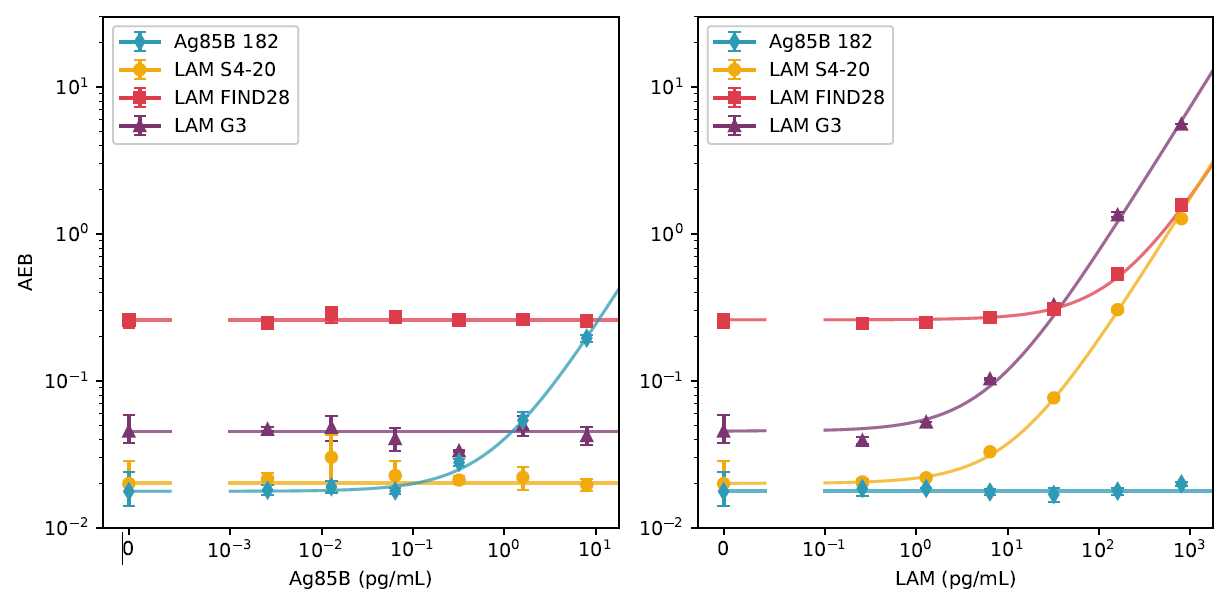


**Figure S3.** No cross-reactivity between the assays in dropout experiments. (Left) The presence of Ag85B did not affect the measured concentration of LAM. (Right) The presence of LAM did not affect the measured concentration of Ag85B.


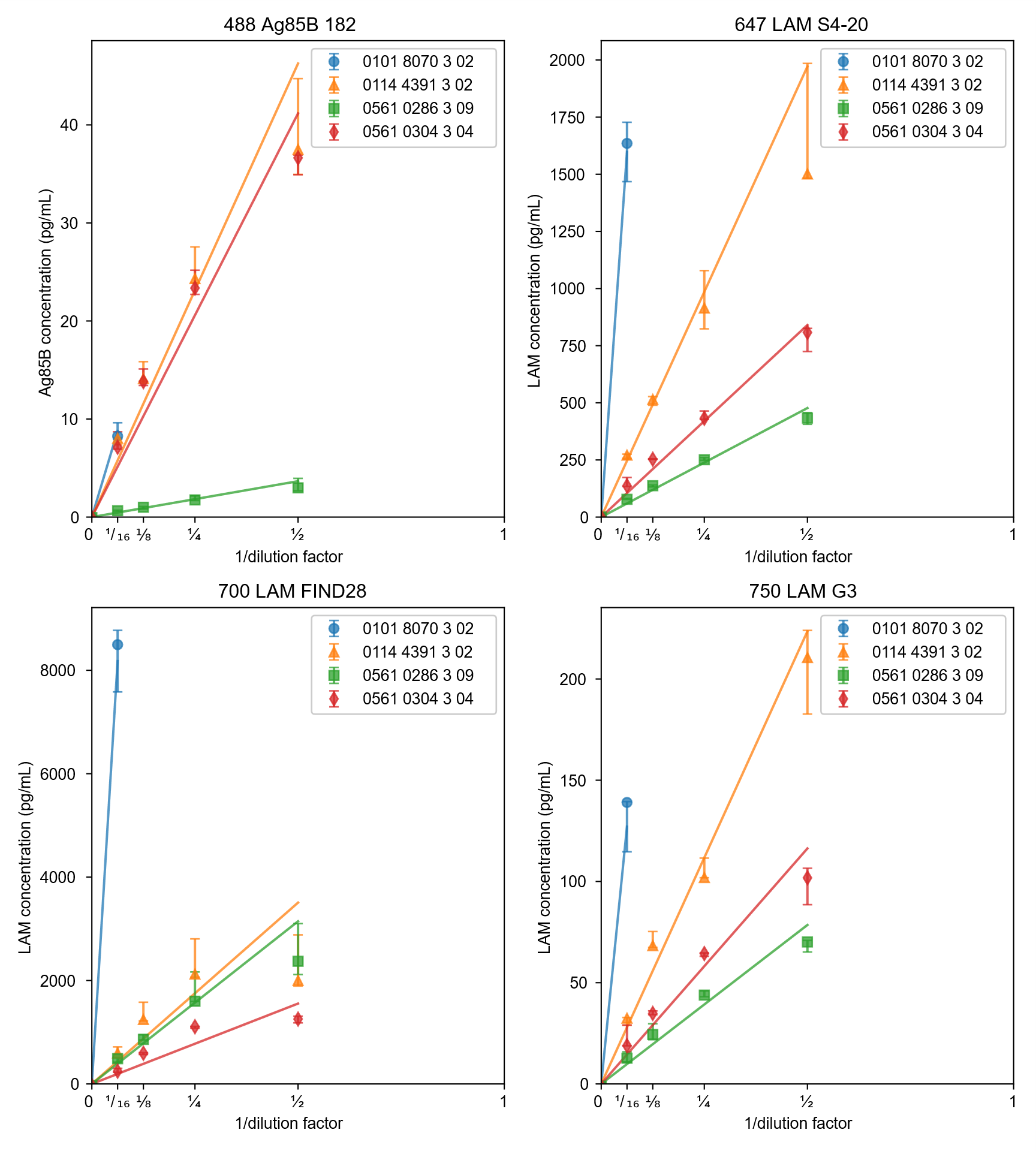


**Figure S4.**Dilution linearity. Urine samples (samples barcodes appear in the legend) were diluted 2X, 4X, 8X, and 16X with sample diluent.


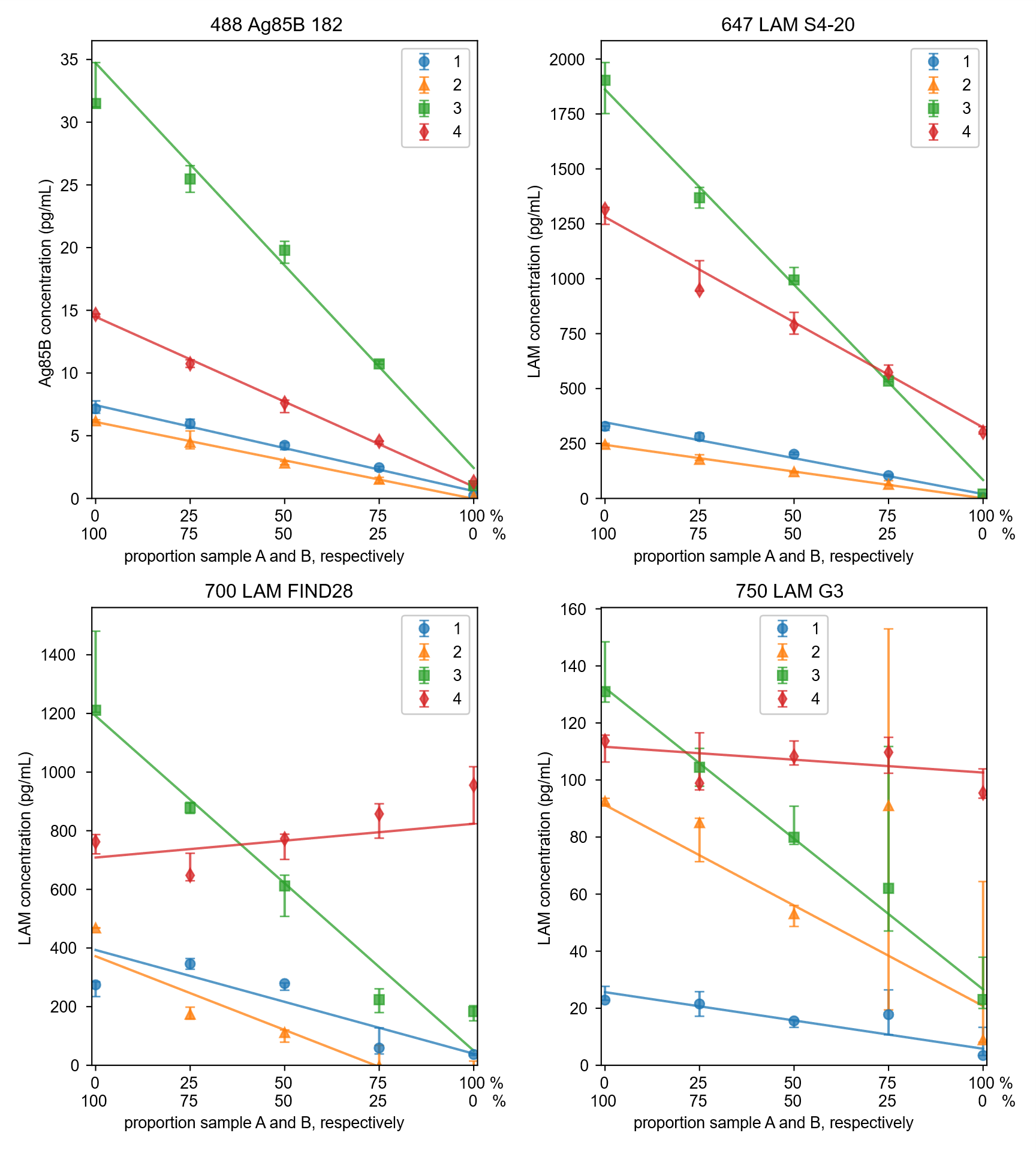


**Figure S5.** Admixture linearity. Urine samples were mixed according to the percentages indicated on the X-axis. Sample barcodes for the mixtures are as follows: 1- Sample A (01014262301), Sample B (05010163303); 2- Sample A (01610257301), Sample B(01610436301); 3- Sample A (01610485301), Sample B(01144309303); 4- Sample A (01610462301), Sample B(01610755301).

**Ag85B pull-down and silver staining**

To verify that the Ag85B capture antibody (182λ) used in this study identifies the native form of Ag85B, we conducted a pull-down assay using cell lysate from the BCG strain. It should be noted that there is a single amino acid substitution in the BCG copy of *fbpB* (expressing Ag85B protein) compared with the reference H37Rv sequence. A volume of 250 µL of each sample or RIPA buffer was mixed with 40 million 488-dyed magnetic beads conjugated with the anti-Ag85B antibody (182λ). Following an overnight incubation with rotation at 4°C, the samples were washed twice with RIPA buffer. Then, the beads were mixed with 3 µL of 500 mM DTT, 7.5 µL of 4X Laemmli buffer, and 19.5 µL of RIPA buffer. Following incubation of the beads at 95°C for five minutes, supernatants were loaded on a 4–12% precast gel (Novex™ WedgeWell™ 4–12% Tris-Glycine Gel) and run for one hour at 150V. The gel was silver stained according to the manufacturer’s protocol (Pierce Silver Stain Kit, 24612). The Developer working solution was incubated for one minute to achieve the desirable protein band intensity. The gel was imaged using BioRad Gel Doc™ EZ imager.

Bands corresponding to Ag85B molecular weight (34.58KDa) are seen only in the positive control lane and the wild-type BCG cell lysate (marked in red rectangle), meaning that antibody-coated magnetic beads pulled down Ag85B from these lysates. These results show that the antibody identifies the native form of Ag85B. There is no band in the negative controls: Ag85B knockout, Ag85C, and without cell lysate. To differentiate the antibody light chain from Ag85B, the 182λ antibody was also run on the gel.


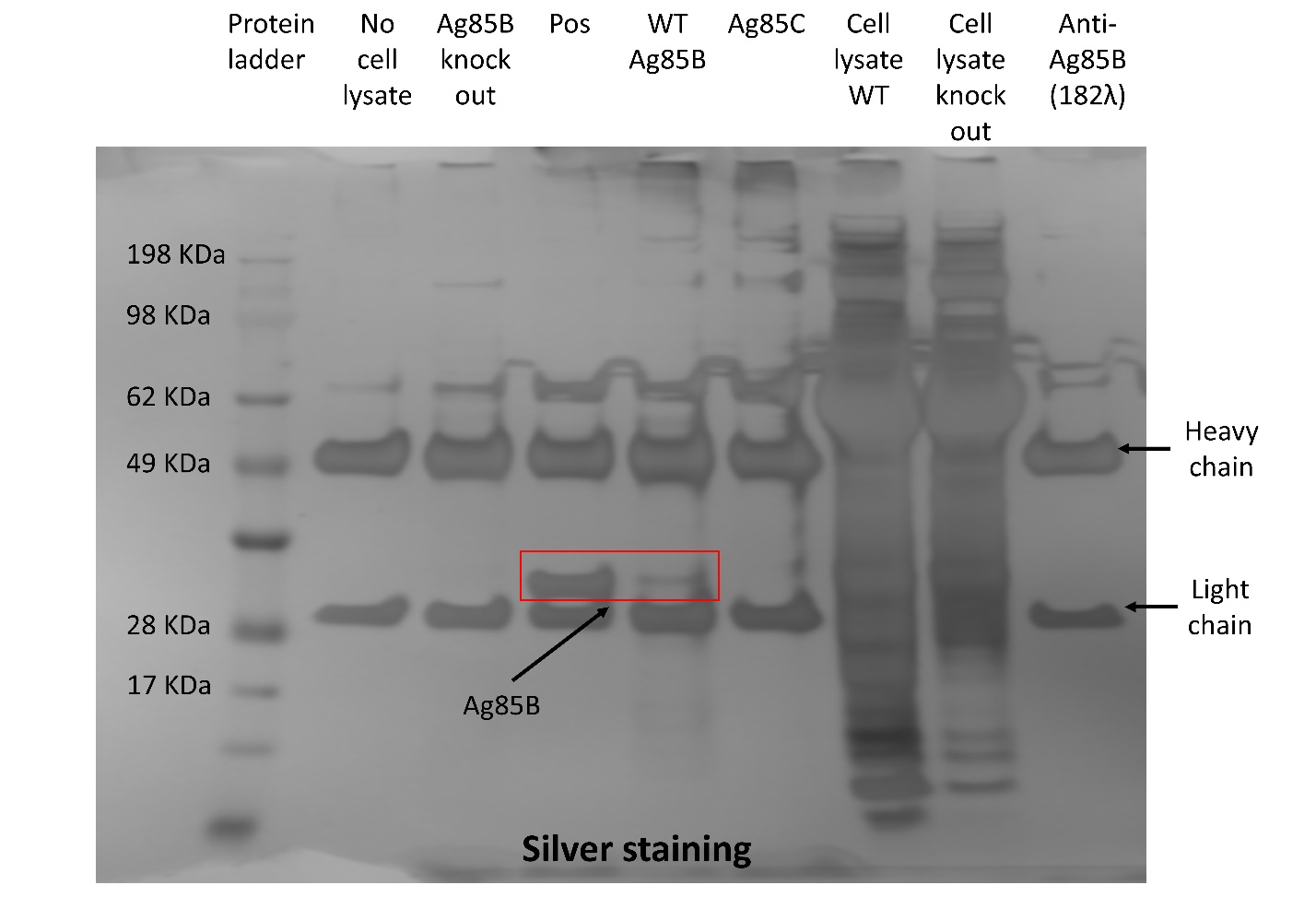


**Figure S6.** Ag85B capture antibody (182λ) identifies the native form of Ag85B. Silver stain of proteins pulled down with Ag85B capture antibody (182λ). Abbreviations: Pos, native Ag85B BEI; WT Ag85B, wild-type BCG cell lysate.


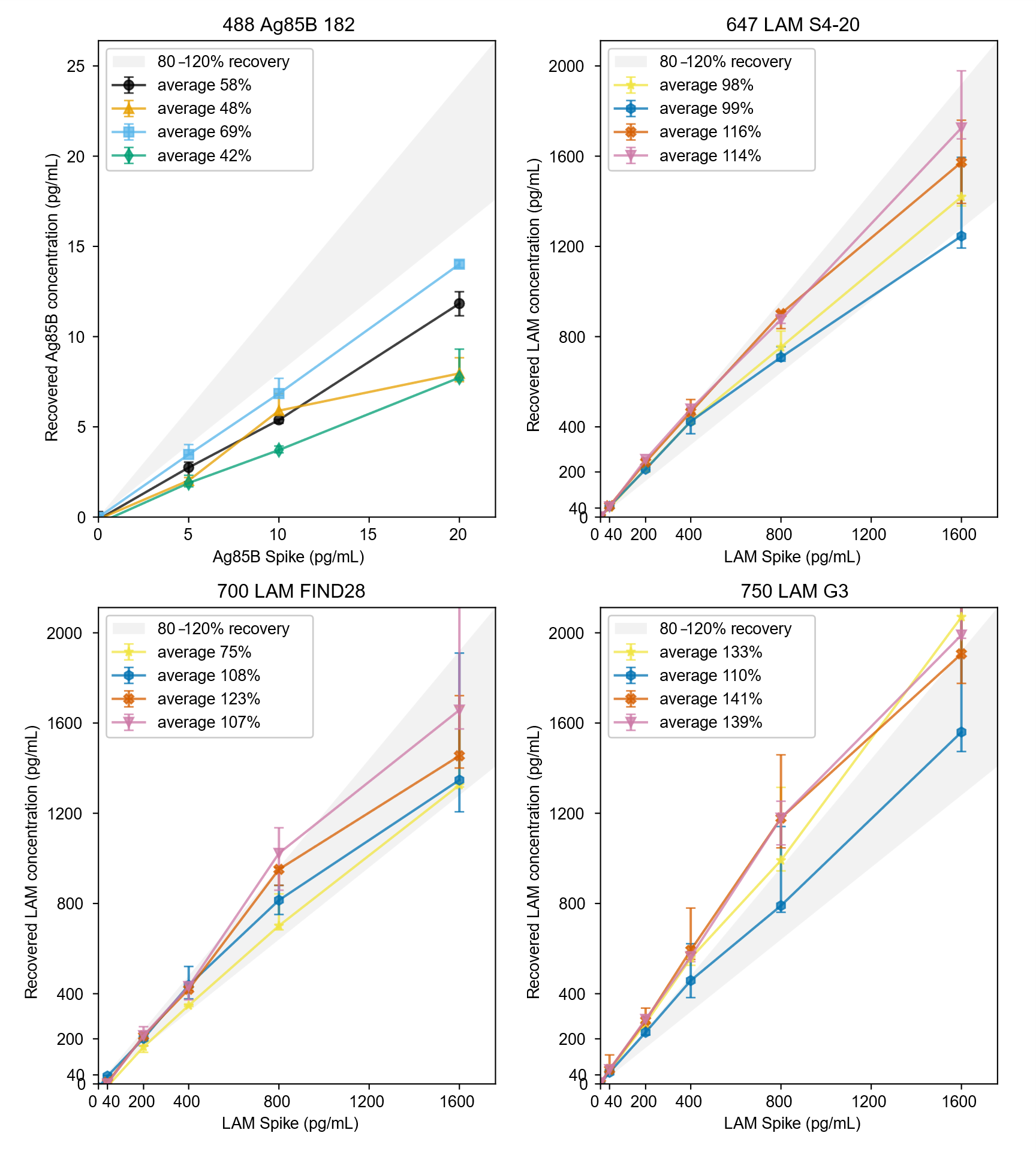


**Figure S7.** Spike and recovery of LAM and Ag85B. Known concentrations of Ag85B and LAM were spiked into negative urine samples from the training cohort. Recoveries were calculated as follows: $\% Recovery=100\%\times\frac{Observed concentration-Endogenous concentration}{Spiked concentration}$.


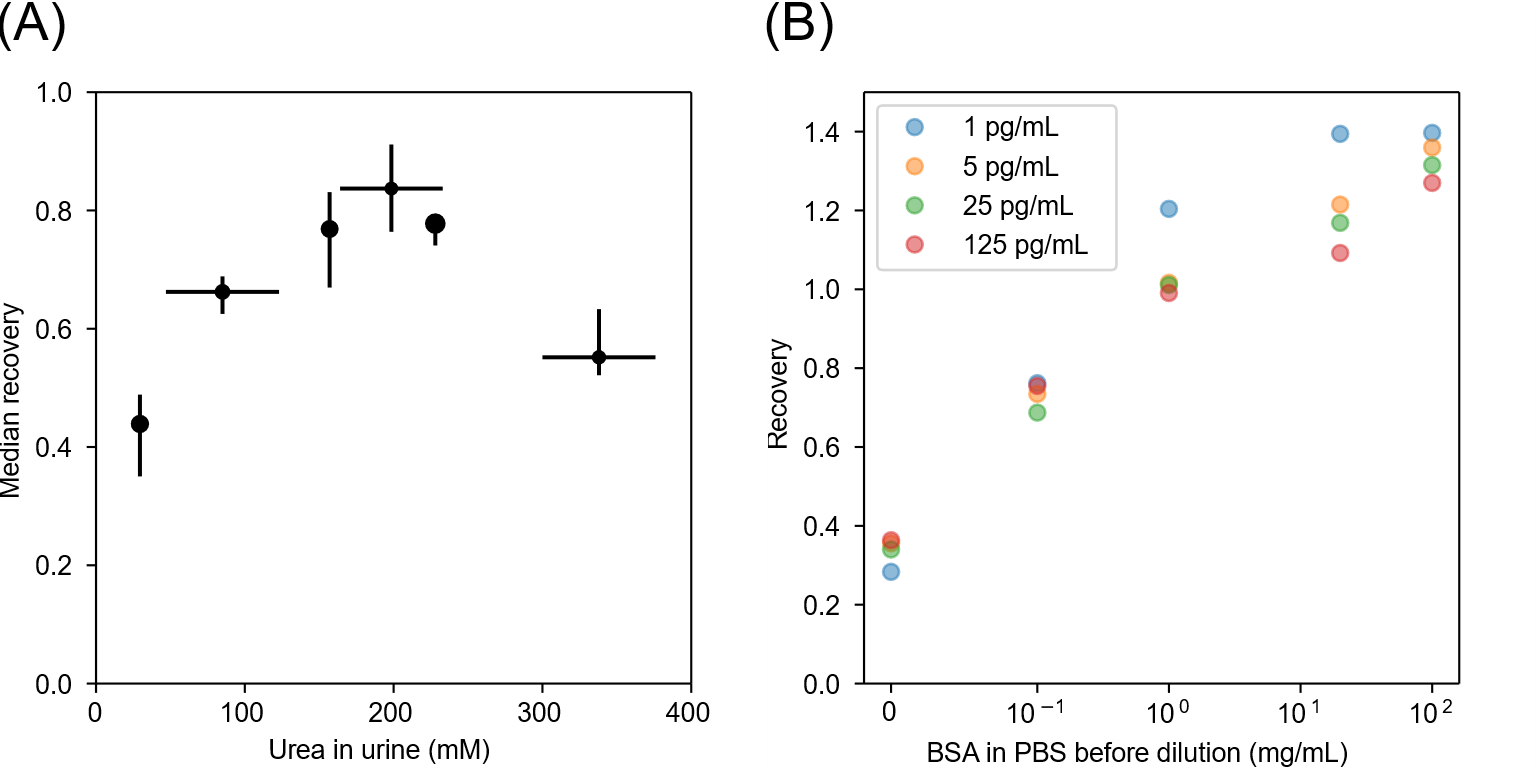


**Figure S8.** Recovery of Ag85B is negatively affected by urea in the urine and is dependent on urine total protein. (A) Urea concentrations in urine were measured using a Urea Nitrogen (BUN) Colorimetric Detection Kit (#EIABUN, Invitrogen). Recoveries were calculated as follows: $\% \mathrm{Recovery}=100\%\times\frac{Observed concentration-Endogenous concentration}{Spiked concentration}$. In the range of the urea tested, corresponding to physiological urea concentrations, reduced recovery was observed. (B) Different concentrations of Ag85B were spiked in PBS containing elevated concentrations of BSA. Without any BSA in the PBS to which Ag85B was spiked in, all spiked Ag85B concentrations resulted in poor recovery of ~ 35%.


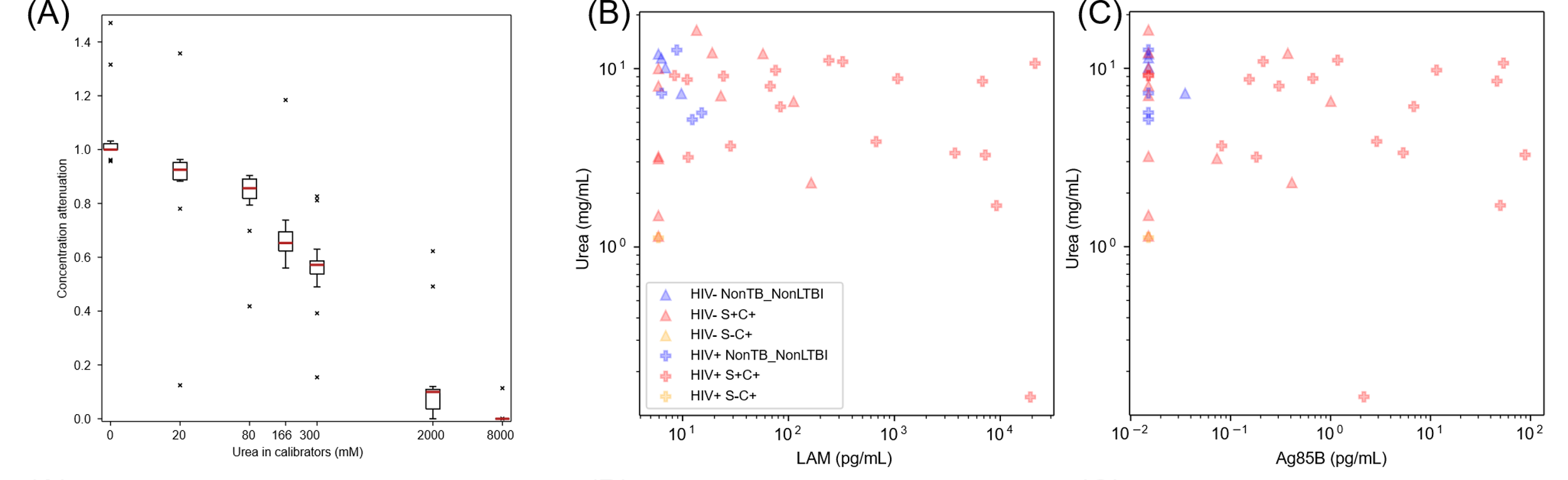


**Figure S9.** Urea attenuates Ag85B concentration and spans a wide range in urine samples. (A) Adding urea to the calibration curve attenuates Ag85B concentration. (B), (C) Urea concentrations in 40 samples from the training and validation cohorts, measured using a Urea Nitrogen (BUN) Colorimetric Detection Kit (#EIABUN, Invitrogen). Urea concentrations range between ~17–166 mM across all LAM and Ag85B concentrations.


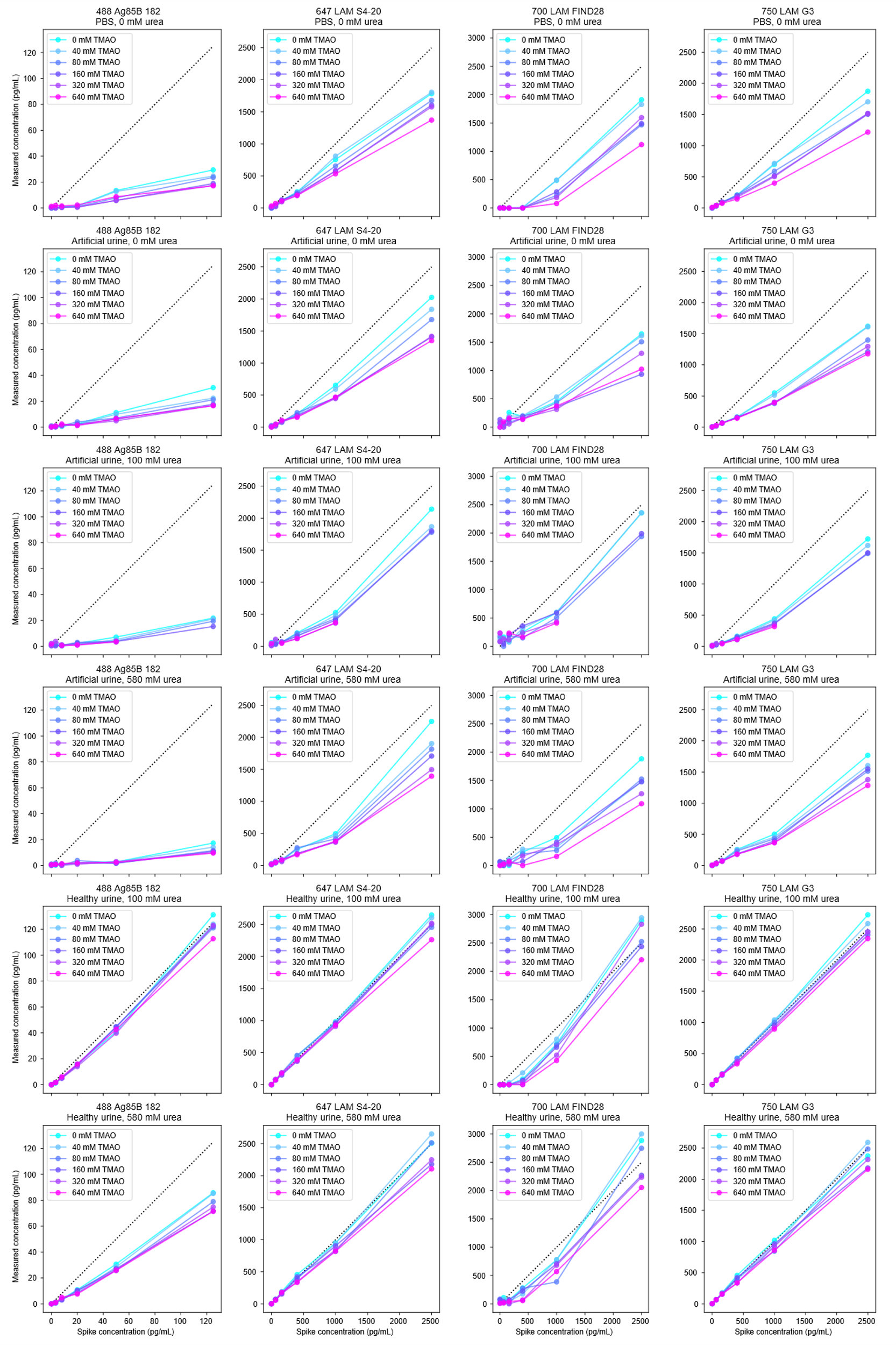


**Figure S10.** Attempts to improve recovery. Adding urea to the spiked matrix slightly improved the recovery of Ag85B. For example, 0 mM TMAO in 100 mM urea in artificial urine compared to 0 mM TMAO in 0 mM urea in artificial urine (light blue curve). The addition of TMAO did not yield higher recoveries. Lower recoveries are seen at the highest TMAO concentrations.

**Figure S11.** Flow diagram of model training and evaluation. Lavender: training and validation cohorts; peach: blinded test cohort; green: model trained on training/validation cohorts and evaluated once on blinded test cohort; grey: models trained and evaluated through nested cross-validation on entire cohort. Widths are proportional to the numbers of samples used in each step. Arrows indicate the flow of data through the study, e.g., the green model is trained on the training/validation cohorts and used to predict the blinded test cohort, which is then unblinded for evaluation, whereas the black models use all samples, with each sample allocated to training or evaluation within each outer fold of nested cross-validation.


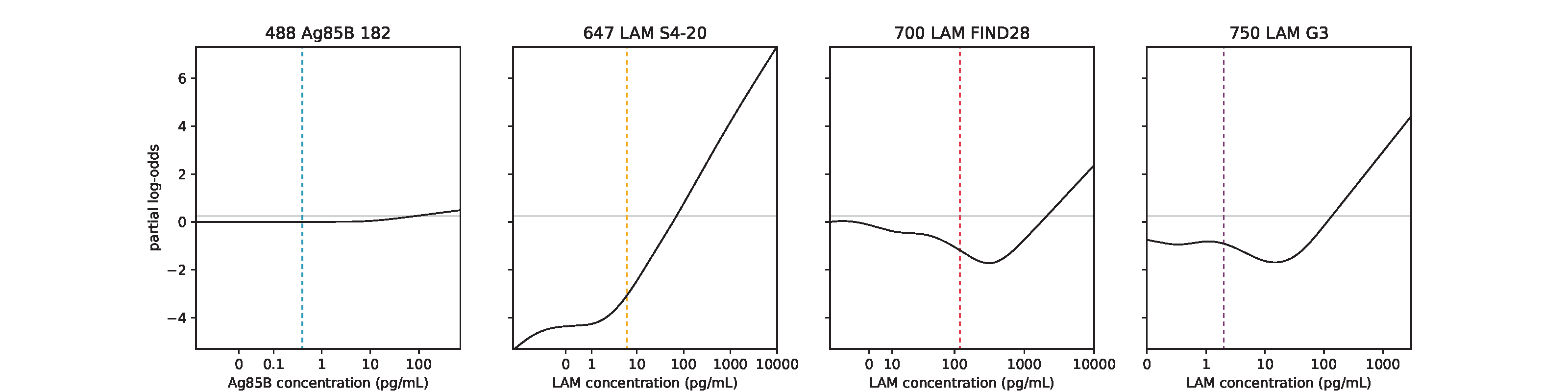


**Figure S12.** Partial dependence plots for the model evaluated on the test cohort. Both axes are scaled due to transformations. Each of these four plots consists of a univariate spline model, and the log-odds assigned to a sample is the sum of all four. The predicted probability score between 0 and 1 is related to this log-odds by a logistic link function.


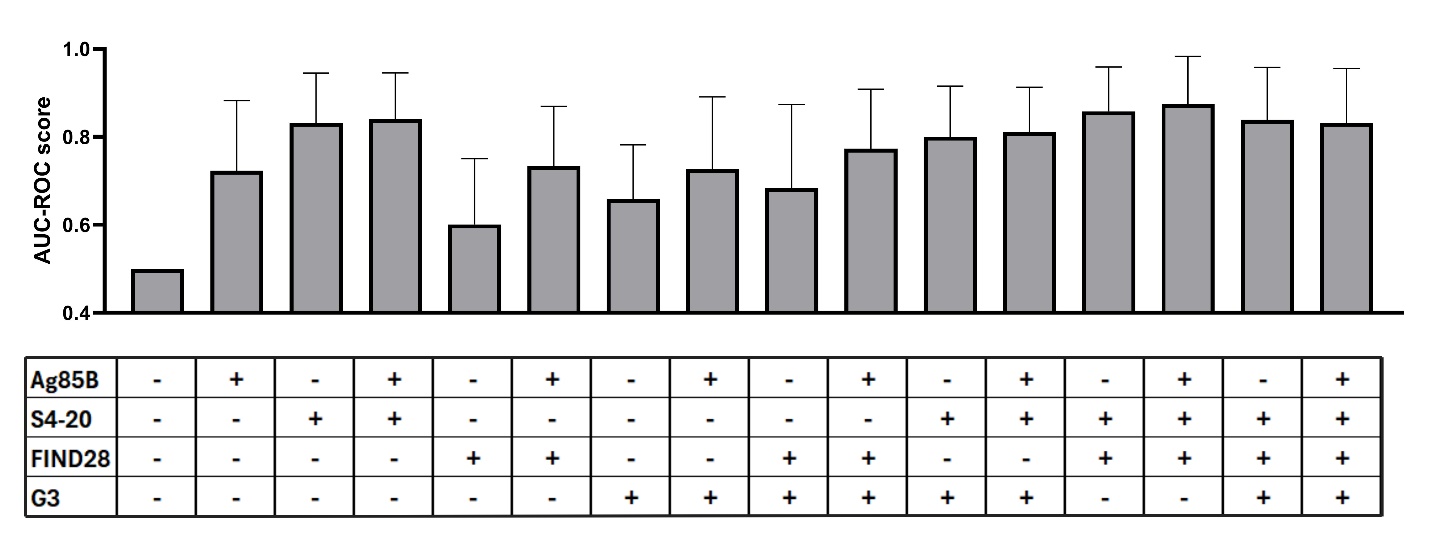


**Figure S13.** AUC-ROC scores with different combinations of biomarkers. The AUC-ROC scores for each individual biomarker and different combinations were calculated. Adding Ag85B to all other combinations improved the AUC-ROC scores.

**Table S4**. Characteristics of common TB diagnostic tests. Red = poor, orange = marginal, yellow = acceptable, green = good, grey = variable or unknown.

|  | **Sample type** | **Assay time** | **Time to result** | **Specificity** | **Sensitivity, S+HIV–PTB** | **Sensitivity, S–HIV–PTB** | **Sensitivity, HIV+PTB** | **Current usage** |
| --- | --- | --- | --- | --- | --- | --- | --- | --- |
| **Clinical** | Signs and symptoms | 10 minutes | <30 min | Variable | Variable | Variable | Variable | Widespread |
| **Radiography** | Chest X-ray | 15 minutes | <30 min | Variable | Variable | Variable | Variable | Used in some contexts |
| **Smear microscopy** | Sputum | 20 minutes | <30 min | 98% (N=5617) (48) | 100% (by definition) | 0% (by definition) | 34% (N=1339) (49) | Widespread |
| **AlereLAM** | Urine | 25 minutes | <30 min | 91% (N=2172) (7) | 16% (N=68) (36) | 2% (N=43) (36) | 42% (N=2172) (7) | Used in HIV+ contexts |
| **Truenat MTB** | Sputum | 1 hour | 1–3 h | 98% (N=1093) (32) | 91% (N=177) (32) | 36% (N=86) (32) | Not tested | Beginning rollout |
| **Truenat Plus** | Sputum | 1 hour | 1–3 h | 96% (N=1093) (32) | 96% (N=177) (32) | 47% (N=86) (32) | Not tested | Beginning rollout |
| **Simoa** | Urine | 1 hour 18 minutes | 1–3 h | 98% (N=576) | 48% (N=91) | 13% (N=39) | 45% (N=576) | Lab research only |
| **Xpert MTB/RIF** | Sputum | 2 hours | 1–3 h | 98% (N=598) (31) | 99% (N=598) (31) | 61% (N=379) (31) | 75% (N=635) (31) | Increasingly widespread |
| **Xpert Ultra** | Sputum | 2 hours | 1–3 h | 96% (N=1851) (31) | 99% (N=593) (31) | 78% (N=378) (31) | 88% (627) (31) | Increasingly widespread |
| **EclLAM** | Urine | 2 hours 10 minutes | 1–3 h | 98% (N=261) (36) | 84% (N=68) (36) | 19% (N=43) (36) | Not tested | Lab research only |
| **Culture (liquid)** | Sputum | 13 days | 2–4 weeks |  | 100% (by definition) |  |  | Confirmatory |
| **Culture (solid)** | Sputum | 26 days | 2–4 weeks |  | 100% (by definition) |  |  | Confirmatory |

**Table S5.** TB categories and classifications. Blank cells indicate cases where the category includes both negatives and positives.

|  | Non-TB, Non-LTBI | Non-TB LTBI | Likely Sub-clinical TB | Clinical TB | S–C+ | S+C+ |
| --- | --- | --- | --- | --- | --- | --- |
| Culture | **–** | **–** | **–** | **–** | **+** | **+** |
| Follow–up culture | **–** | **–** | **+** |  |  |  |
| Smear | **–** | **–** | **–** | **–** | **–** | **+** |
| Xpert | **–** | **–** | **–** | **–** |  |  |
| CXR abnormalities |  | **-** |  | **+** |  |  |
| Symptoms | **+** | **+** | **+** | **+** | **+** | **+** |
| QuantiFERON-TB Gold (QFT) | **–** | **+** |  |  |  |  |
| Started on Treatment | **–** | **–** | **–** | **+** | **+** | **+** |
| Classification | **-** | **-** | **-** | **+** | **+** | **+** |

**Table S6.** Alere LAM results using 244 samples.

|  | **Alere LAM** | **FIND** |
| --- | --- | --- |
| **Positive** | 22 | 58 |
| **Negative** | 222 | 186 |
| **TP** | 7 |  |
| **FN** | 51 |  |
| **TN** | 171 |  |
| **FP** | 15 |  |
| **Sensitivity** | 12% |  |
| **Specificity** | 91.9% |  |

**References:**

48. Davis, J.L., Cattamanchi, A., Cuevas, L.E., Hopewell, P.C. & Steingart, K.R. Diagnostic accuracy of same-day microscopy versus standard microscopy for pulmonary tuberculosis: a systematic review and meta-analysis. *The Lancet Infectious Diseases* **13**, 147-154 (2013).

49. Broger, T.*, et al.* Diagnostic yield of urine lipoarabinomannan and sputum tuberculosis tests in people living with HIV: a systematic review and meta-analysis of individual participant data. *The Lancet Global Health* **11**, e903-e916 (2023).
